# Left in the lurch: a junior doctor's scathing critique of the Nigerian healthcare system

**DOI:** 10.11604/pamj.2024.49.28.45301

**Published:** 2024-10-04

**Authors:** Muhammed Raji Modibbo

**Affiliations:** 1 Department of Medicine, Federal Medical Centre, Jabi, Abuja, Nigeria

**Keywords:** Nigeria, public health, administration, healthcare policy, junior doctor

## Abstract

This reflective article examines the profound challenges faced by junior doctors in Nigeria, focusing on the pervasive lack of support from senior colleagues and the systemic failures within the healthcare system. Drawing from personal experiences, the narrative highlights how newly qualified doctors are often “left in the lurch”, thrust into demanding roles with insufficient guidance, training, and resources. The consequences of this abandonment are explored, not only in terms of the personal and professional toll on junior doctors but also in the broader context of patient care and the overall efficacy of the healthcare system. The article concludes with a call for urgent reforms to address these critical issues, advocating for a more supportive and sustainable environment for the next generation of medical professionals in Nigeria.

## Essay

**Systemic failings:** the sound of my ringtone woke me up after only a few minutes of sleep. It was 2:30 a.m, and there was another emergency. I was the only doctor on duty, with no senior in sight, and a hospital full of patients. The harsh reality of being a junior doctor in Nigeria hit me like a ton of bricks. I was completely on my own.

The major driving force behind medical emigration from Nigeria is the pursuit of a better salary [1]. From students to seasoned consultants, everyone is saving up for foreign exams, all in search of more rewarding compensation. What other factor could there be when we´re talking about professionals at opposite ends of the career spectrum? The common thread binding them is the promise of a more generous paycheck. Yet, this problem is not isolated to the medical sector alone; it permeates every facet of society. Every citizen yearns for higher wages, a stable economy, and a safer nation. Nigeria´s challenges are obvious and addressing them would go a long way in retaining our most skilled workers. It is up to our leaders to fix these issues, and in this regard, the glass isn´t half empty; the glass doesn´t exist. But in Nigeria´s healthcare specifically, our system suffers from systemic inefficiencies, including insufficient government funding, pervasive and staggering corruption, inadequate budget allocation, and a lack of political will to drive improvements. Junior doctors, fresh from school, are expected to navigate this chaotic landscape, often shouldering responsibilities far beyond their experience while contending with a healthcare system that neglects their needs.

In Nigeria, house officers are recent medical graduates with temporary registration from the Medical and Dental Council of Nigeria (MDCN), undergoing a one-year internship program [2]. Upon completion, they receive full registration as medical practitioners. National Youth Service Corps (NYSC) doctors, meanwhile, are junior doctors in their second year of work, following their internship. These early years are marked by overwhelming stress and a lack of proper support. Junior doctors are, quite frankly, left in the lurch, abandoned by a system that demands everything but offers next to nothing in return.

**Personal experience:** to illustrate, let me share a personal anecdote: during my NYSC, I worked in internal medicine; however, due to organizational issues, the hospital administration needed me to cover departments with staff shortages. On my very first night shift, I was left alone to manage the pediatric emergency ward, with a second on-call who “forgot” to answer the phone. A family arrived, wailing and in angst, with a newborn. The baby had been delivered at home, and the mother had not attended any antenatal visits during her pregnancy. The baby had not cried at birth and had not cried since then. They also described unusual movements that I suspected might be seizures. I immediately assumed perinatal asphyxia; I remembered attending a seminar on it before, but managing such an acute case independently was terrifying.

I called for help, and with a team of nurses around me, began resuscitating the baby. As cardiopulmonary resuscitation (CPR) was ongoing, I started making arrangements for an ambulance and then advised the family to transfer the baby to a larger facility with better resources and more capable hands.

However, their frustration and anxiety, understandably, turned into anger. They accused me of incompetence and even threatened physical violence. I tried to explain the limitations of our facility, the need for neonatal intensive care unit (NICU) monitoring, and the honest situation I found myself in. But my pleas fell on deaf ears. To them, the limitations of the system translated into limitations of myself as a doctor. The scene quickly got out of control. Throughout the ordeal, I kept hoping against hope to contact a more senior colleague who could either help me manage the situation or at least quell the tension, but there was no response. I was forsaken. I was thrown to the ground, and a chair was flung in my direction. At that point, I ran from the emergency room, fearing for my safety. I retreated to the doctors' lounge and called the hospital´s compound office for security reinforcement.

Upon reflection, I realized that it did not matter to the family that I had received no formal orientation at the facility. It did not matter that our hospital lacked a functional NICU. It did not matter that I was an internal medicine doctor thrust into a specialty I had no business being in, nor did it matter that, in treating newborns, my experience was limited to a four-week Neonatology rotation during my internship. What mattered to them was the stethoscope around my neck. My circumstances did not matter to them, and honestly, why should they? To them, I was the embodiment of the failures of the healthcare system. I felt used and abused, not by the family that attacked me, but by the administration that put me in that position to begin with.

**The bigger picture:** moments like this, where healthcare workers are put in harm´s way [3,4], make it clear why so many Nigerian doctors are leaving in droves. Working in unsafe environments is untenable. These are not isolated incidents; the constant battle with systemic problems, the overwhelming sense of isolation, and the absence of a proper support system are significant reasons why many doctors in Nigeria, myself included, contemplate leaving the country. While the pursuit of financial security is undeniably a driving factor, the desire to practice medicine in a nurturing environment that provides the resources, support, and security our profession demands is equally, if not more, important. In nearly all parts of the world, junior residents have a senior available. Unfortunately, this is not the case in Nigeria. The most vulnerable members of the medical community are often thrown into the deep end with zero lifelines. On-call rotas in Nigeria are designed to cover gaps rather than considering the quality of healthcare delivery, the training of young doctors, or their well-being. Of course, one reason rotas cover gaps in this manner is the severe shortage of doctors in Nigeria. The country faces a critical deficit of skilled healthcare workers, with doctor-patient ratios that are 15 times worse than the WHO-recommended standard [5]. This shortage leads to overburdened staff and compromised patient care. It also causes significant trauma for healthcare professionals who are inadequately supported [6].

In saner climes, emergency medicine is a specialty unto itself, with board-certified consultants overseeing the emergency room (ER). In Nigeria, however, 12 months post-university is considered sufficient experience to handle any case the ER has to throw at you. There are barely any advanced cardiac life support (ACLS) training programs in Nigeria, there are no defibrillators in our ERs, and there are no safety checks at all. Junior doctors are the first to bear the brunt of these inadequacies. The only junior doctors who thrive in this environment are those who have mastered the art of “looking busy”, “Showmanship,” as it is called. It is obscene. Our system raises significant concerns about the safety and quality of care provided to Nigerian citizens.

**A call for reform:** how can we curb the medical brain drain, you ask? Firstly, hospitals should have better amenities for doctors, including adequate security to de-escalate workplace violence. Secondly, there should be emergency training requirements for junior doctors before they assume duty. Thirdly, emergency equipment must be provided in every ER across the country. Furthermore, hospitals should not be allowed to move doctors from their areas of training just to fill roles the hospital needs. We all know medicine is a career that requires on-the-job learning, but there are safer ways to go about it. It is often said that manning the ER alone is how you gain experience, and while I agree, I have to ask: how many people, doctors and patients alike, need to be sacrificed at the altar of this experience?

I've heard stories from colleagues who were deployed to rural areas across the country during their NYSC, serving as the only doctor in the community. They´ve told me about performing C-sections and appendectomies, unaided and unassisted. Many of them seem proud of these feats. As for me, I´m not sure who I pity more my colleagues or the patients they attended to.

My experience is emblematic of the kind of environment that encourages doctors to leave Nigeria. The healthcare system, guided by the corrupt hand of the political class, is driving its own people away. When young doctors receive no support and are forced to work in unsafe environments with inadequate resources, while their well-being is constantly overlooked, the only viable option for many is to seek opportunities elsewhere. Medical emigration is not just about better pay or living conditions; it is about the opportunity to practice medicine in a system that values its doctors and enables them to provide the best possible care. Unless urgent reforms are made, Nigeria will continue to lose its brightest minds to countries that offer them the dignity and respect they deserve. We need to do better. We need to protect our doctors better, train our doctors better, and serve our people better.

## References

[1] Ramalan MA, Garba RM (2021). Determinants of Nigerian Medical Doctors' Willingness to Practice in Foreign Countries. Nigerian Journal of Medicine.

[2] Alozie N, Egbuchulem KI, Okor MC, Akintepede F, Omogiade CA, Awodiji MM (2023). The ideal house officer: trainee's perspectives. Ann Ib Postgrad Med.

[3] Adejoro L (2024). 146 resident doctors assaulted in two years - Report..

[4] Onwuzoo A (2023). Enact laws to protect health workers against vicious attacks. doctors tell National Assembly.

[5] Aderinto N, Kokori E, Olatunji G (2024). A call for reform in Nigerian medical doctors' work hours. Lancet.

[6] Salako P (2023). Death on duty: State negligence leads to exodus of Nigerian doctors.

